# Orthodontic management of traumatic avulsion of permanent incisors in a child with sickle cell anaemia: a case report

**DOI:** 10.4076/1757-1626-2-8123

**Published:** 2009-08-26

**Authors:** Sanu O Oluwatosin, Oredugba A Folakemi, Temiye O Edamisan

**Affiliations:** 1 Orthodontic Unit, Department of Child Dental Health, College of Medicine, University of LagosLagosNigeria; 2 Paedodontic Unit, Department of Child Dental Health, College of Medicine, University of LagosLagosNigeria; 3 Department of Paediatrics, College of Medicine, University of LagosLagosNigeria

## Abstract

**Introduction:**

Avulsion of permanent teeth in children resulting from trauma is an acute emergency presentation. When not adequately managed, it may result in functional and aesthetic disturbances, with implications for more complicated and prolonged treatment which require planning and biocompatibility in terms of forces used in moving the teeth orthodontically. Literature is scarce on a reported case of this nature and this is the first report in the literature in Nigeria.

**Case presentation:**

We report on a case of avulsion of both maxillary central incisors in an 8-year-old male child of Sub-Saharan African ethnicity with sickle cell anaemia. One of the incisors produced by the parents was replanted at the clinic. The replanted tooth was later traumatized during the course of treatment which resulted in mobility and subsequent extraction of the tooth. At a later presentation at the clinic, orthodontic therapy was instituted. While orthodontic therapy has been considered to be completely noninvasive, special precautions should be taken in the clinical management of sickle cell anaemia patients to prevent local vaso-occlusive events.

**Conclusion:**

The sequeale of traumatic avulsion in a medically compromised patient with sickle cell anaemia is presented. Prompt and early presentation for dental management is very important; while prevention and management of dental trauma should be recognized as a public health issue. Dental trauma in sickle cell anaemia can be minimized by practicing preventive measures with the use of mouth guard which is an effective device for preventing dental injuries, and patients should be advised to wear them during activities to prevent dental injuries.

## Introduction

Traumatic injuries of permanent incisors and their supporting structures constitute a true dental emergency and require immediate assessment and management [1]. Avulsion of permanent teeth occurs most often in 7 to 9 year old and the maxillary central incisors are the most commonly affected [2].

Traumatic injuries affecting both the primary and permanent dentitions and their supporting structures are a common problem seen in children [3]. Although such trauma does not represent a significant morbid risk for the patient, it can result in functional and aesthetic disturbances with concern of patients and parents [3]. The patients, who are exposed to trauma, are not only physically, but also psychologically affected. The consequences of trauma vary from tiny fractures to the complete avulsion of the tooth [4]. Successful management of the avulsed tooth however begins at the accident site; correct first-aid care followed by proper management in the dental surgery will significantly improve the prognosis of the avulsed tooth [3].

The prognosis of traumatized teeth depends on prompt and appropriate treatment, which often relies on lay people around the child at the time of the accident. These are usually the children’s parents and their schoolteachers who are present at the site of accident prior to the initial professional dental contact [5].

In a study in Nigeria, most parents were not confident about carrying out replantation of avulsed tooth into its socket and in fact do not know how to do it [3]. The responses obtained in the study included brushing an avulsed tooth with a toothbrush, a procedure that might disrupt the periodontal ligament cells on the tooth surface, severely decreasing the chance of successful replantation [3].

Long-term sequelae include shifting of remaining teeth with resulting misalignment and periodontal disease. There is redistribution of spaces in the anterior region as a result of early or premature loss of permanent teeth into spaces created by the missing teeth. There is associated embarrassment which affect the quality of life of the patient.

Acute infections of dental origin are a well-known trigger of sickle cell crises and should therefore be prevented [6]. Piccin A et al. [6] reported that dental infection when it occurs must be promptly and adequately treated. Individuals with sickle-cell disease are more prone to developing osteomyelitis because of hypovascularity of the bone marrow secondary to thromboses [7].

The use of local anaesthesia is not contraindicated in patients with sickle-cell disease. Lignocaine with adrenaline 1:80,000 can be used safely [8].

The prevalence of patients with underlying medical conditions seeking orthodontic care has increased over the past few decades. This means that increasingly, orthodontists are treating medically compromised patients [9].

Literature is scarce on a reported case of this nature. We present a case of trauma resulting in avulsion of the anterior teeth in a sickle-cell patient. Orthodontic and restorative management of the case is presented.

## Case presentation

An 8-year-old male child of Sub-Saharan African ethnicity presented to the Paediatric Dentistry Unit of Lagos University Teaching Hospital, Lagos, with a history of trauma sustained in an accident. The accident occurred while playing at school a day prior to his presentation at the dental clinic. It resulted in avulsion of both maxillary central incisors at the time of the accident. There was minimal bleeding.

The maxillary left central incisor found at the site of the accident, was kept in a cup of milk and brought along to the clinic the next day at the time of presentation. However, the maxillary right central incisor could not be found.

His medical history was contributory as the patient was diagnosed with SCA prior to presentation. The estimated haemoglobin percent was 4.5 g/dl, with several sickled red blood cells in the peripheral blood smear. Haemoglobin electrophoresis revealed “haemoglobin S” which was diagnostic of HbSS.

After gently irrigating the socket for foreign bodies, an attempt was made at replantation of the maxillary left central incisor. Splinting was done with 0.018 inches stainless steel orthodontic wire bonded to the teeth with orthodontic adhesive. The patient was started on medications-Amoxicillin (50 mg/kg 8 hourly daily for 5 days) and given tetanus toxoid, after a stat dose for sub acute bacteria endocarditis (SBE) and tetanus prophylaxis respectively. Paracetamol tablets (500 mg 8 hourly for 3 days) were given for analgesia and a chlorhexidine mouth wash used for 1 minute twice a day.

Patient was reviewed three days after and was reported to have bled moderately from the socket of the replanted tooth. The patient and parents were reassured and the patient was asked to be reviewed after one week.

**Figure 1.**
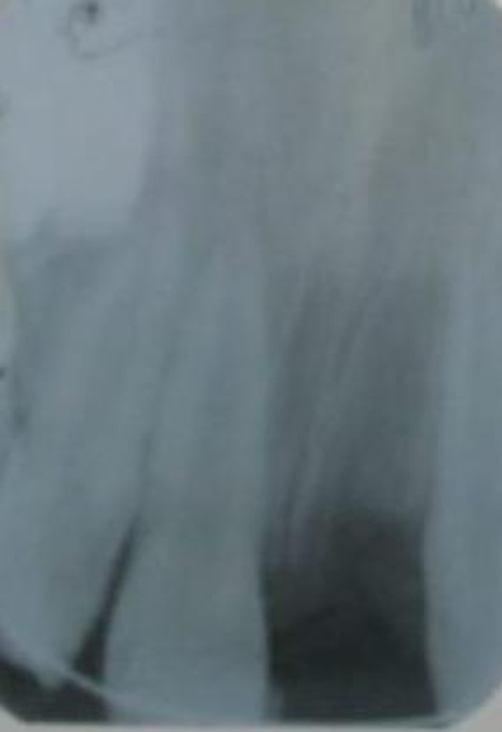
Periapical radiograph after replantation of maxillary left central incisor. Notice the orthodontic wire used for splinting.

At ten days post splinting, a radiographic examination of the maxillary anterior region was taken (Figure 1). The patient and parent were given instructions as to the importance of good oral hygiene for preserving oral health and the patient was instructed not to tear off food with the anterior region.

However, at about two weeks after the splinting, he was involved in another domestic accident at home and sustained another injury to the replanted tooth, resulting in gross third degree mobility of the tooth. Replantation of the tooth was deemed to have failed and the tooth was eventually extracted and denture fabrication suggested because of the effect on aesthetics. However, the patient was lost to follow up and was not seen again until 4 years after the accident by which time there was total loss of space for the maxillary central incisors. The mandibular incisors had supraerupted and were mildly crowded. A referral was made for an orthodontic assessment.

Extra-oral assessment showed the patient had a mild maxillary protrusion associated with increased medullary activity. There was no circumoral musculature strain on lip closure. He had a high lip line with 2 mm gingival display on smiling.

Intra-orally, he was in the permanent dentition stage with the exception of the third molars. He had an unrestored dentition and was caries free. His oral hygiene was fair.

There was total loss of space for maxillary central incisors.

In the mandibular arch there was mild crowding of the lower labial segment with exaggeration of the curve of Spee. The mandibular incisors were supraerupted into the space in the maxillary arch due to loss of the central incisors. The buccal segments were reasonably well aligned. In the maxillary arch there was total loss of central incisors space, however the buccal segments were reasonably well aligned.

In occlusion, he had a Class II division 1 incisor relationship with minimal overjet and a 5 mm complete overbite. The molar and canine relationships were in Angle’s Class I bilaterally.

Aims of treatment included:

- Creation of space for maxillary central incisors.
- Relief of crowding, alignment and leveling of the lower anterior segment.
- Replacement of maxillary central incisors with removable prosthesis.
- Achieve good functional, as well as stable occlusion

### Orthodontic management

**Figure 2.**
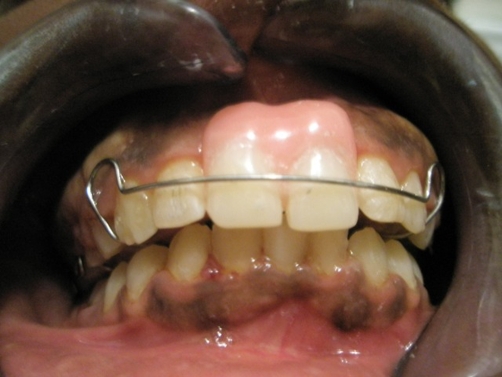
Clinical photograph of the patient at completion of treatment. Note the Hawley Retainer with stock teeth (maxillary central incisors) incorporated.

Pre-adjusted Edgewise brackets (0.022 × 0.028 inch slot, Roth prescription) were bonded to the maxillary and mandibular teeth. Extraction of the lower left lateral incisor was done to relieve crowding in the lower arch and for leveling the lower anterior segment. Space was regained in the maxillary anterior segment progressively using stainless steel open coil springs. The space measured approximately 12 mm, four months into treatment, and about 20 mm, 10 months into treatment. A passive coil spring was placed subsequently to maintain the space gained. By 12 months into the treatment, there was adequate space gained to accommodate the maxillary central incisors. After a review by a paediatric dentist (FAO), a partial acrylic denture was planned to replace the lost teeth. Lower arch leveling and alignment continued until the lower arch was well aligned. After 18 months into treatment he was finally debonded and a Hawley retainer with stock teeth incorporated was fitted. Patient and parents were satisfied with the outcome of the treatment and the subsequently improved aesthetic (Figure 2).

Throughout the period of orthodontic treatment, the patient did not present any vaso-occlusive crisis.

## Discussion

The maxillary central incisors are the teeth most affected by oral traumas [10,11]. This is similar to findings in other studies. The age of occurrence of the patient in this report is when most injuries occur. It was reported that the most accident-prone age is between 8 and 10 years [12]. Local and international surveys reported that males experienced significantly more dental trauma to the permanent dentition than females [13]. In this case report, the patient is a male and is therefore more susceptible to trauma. Most boys also take part in contact sports and are more adventurous than females. Males were reported to be twice as often injured as girls with a ratio of 2.3:1 [12].

The accident occurred at school and is in agreement with an earlier study that reported accidents at home and school account for most injuries to the permanent dentition [14]. Replantation of the avulsed upper left central incisor was deemed to have failed and had to be extracted. This might have been due to several reasons among which may be prolonged extra-oral time and repeated trauma sustained by the patient. It was reported that successful management of the avulsed tooth begins as soon as possible at the accident site [3]. Extra-oral time is important in determining the success of tooth replantation [5]. Studies have shown that the prognosis of replanted teeth is best if replantation is carried out within 5 minutes after avulsion [3,5]. The patient in this report was not seen until over 24 hours after the accident occurred. A delay in providing emergency dental treatment may jeopardise the prognosis of an avulsed tooth [5].

Where the tooth cannot be replanted immediately, the tooth should be stored in an appropriate medium such as normal saline, milk, saliva, Hanks solution or water [15]. Though the parents admitted that the tooth was kept in milk, it was kept for longer than 3 hours. It was reported that milk is shown to maintain vitality of periodontal ligament cells for 3 hours being relatively bacteria-free with pH and osmolarity compatible with vital cells [3].

Successful replantation of teeth with partially formed roots allows root growth, and the teeth maintain their capacity for functional adaptation. Although the replanted tooth sometimes does not respond to electric pulp testing, endodontic treatment is usually not necessary. In this case, because the root of the maxillary left central incisor was partially formed, endodontic treatment was not carried out. Further insult to the periodontal ligament cells on the root surface of the replanted tooth should be minimized. In the reported case, there was a repeated trauma leading to a total breakdown of the periodontal ligament of the tooth. The tooth became very mobile and was finally extracted.

The patient was lost to follow-up as response to recall and follow-up visit is very poor in this environment. Space was lost in the dental arch as a result of drifting of adjacent teeth. The patient had to undergo orthodontic treatment to regain the space for the avulsed maxillary central incisors. Orthodontic treatment in individuals with SCA is not contraindicated [16]. However, the probability that individuals with SCA may present with poor outcome for orthodontic treatment is high. This is probably attributable to the local vaso-occlusive crises seen in SCA patients, which is due to ishaemic injury to the tissues after the obstruction of small blood vessels by the sickled red blood cells. This prevents the local blood from circulating and leads to local oxygen depletion, acidosis, necrosis, and severe pain [17].

When a patient with SCA needs orthodontic treatment, the practitioner involved should know about the disease and the respective treatment. Complete blood supply after application of intraoral and extraoral forces is important and must be maintained [16].

Special precautions should be taken in their clinical management which in most cases is multidisciplinary involving various disciplines in medicine and dentistry. It was reported that necessary care should also be taken to prevent other infections from contaminating the clinical setting in this group of individuals [16].

Adequate levels of oxygenation and bodily temperature were ensured during the orthodontic treatment, and care being taken to use minimal forces. Undue emotional stress was avoided during orthodontic treatment by scheduling his clinical appointments during the chronic phase of the disease. The patient’s appointments were largely planned for early in the morning. When the patient was in any form of pain on presentation for his orthodontic appointment, he was referred to his physician and his appointment rescheduled as necessary. These measures ensured that the patient did not present in any vaso-occlusive crisis throughout his orthodontic treatment. In spite of reports on the susceptibility of SCA subjects to a variety of infections, Crawford working with American SCA patients reported no significant difference in the severity of periodontal disease compared to non-SCA subjects [18]. In another study in Nigeria, it was shown that SCA does not necessarily lead to increased severity of periodontal disease in homozygous HBSS subjects [17]. However, due to susceptibility to other infections in this environment generally and the compromising health condition of SCA patients, there was need to give an antibiotic cover. Sickle-cell anaemia patients are known to have trouble clearing certain bacteria from the bloodstream. Dental procedures can cause bacteremia and due to enlarged heart to compensate for the chronic anaemia often seen; the endothelia lining of the blood vessels in these individuals is often damaged and inflamed [7]. Therefore, Amoxicillin dosage of 50 mg/kg (maximum of 2 gm as a single oral dose), was given 1 hr prior to dental procedures. There is however no evidence-based guideline for premedication of patients with sickle-cell disease.

Though the mandibular left lateral incisor was asymptomatic, it had to be extracted for orthodontic reason to relieve crowding in the lower anterior segment and to allow leveling of the exaggerated curve of Spee. The lower incisors had supraerupted due to longstanding loss of maxillary central incisors. A complete blood count was requested for preoperatively. The haemoglobin percent was found to be about 10.2 g/dl, with white blood cell count within normal range. Lignocaine with adrenaline 1:80,000 was used and there was no need for blood transfusion.

## Conclusion

Prevention and management of dental trauma should be recognized as an acute emergency. Prompt and early presentation for dental management in cases of avulsion is very important. Complications may result and may involve orthodontic therapy. However, orthodontic therapy must be planned and biocompatible forces used to restore regional microcirculation around the teeth that are being moved. The orthodontist should pay attention to changes in the bone turnover during orthodontic movements. Movements of teeth and the forces applied on them should be reduced. However, intense forces should be carefully planned. Trauma in SCA children can be minimized by practicing preventive measures. A dental mouth guard is an effective device for preventing dental injuries, and patients should be advised to wear them during activities where dental injuries are possible.

## References

[bib-001] Zuhal K, Semra OEM, Huseyin K (2005). Traumatic injuries of the permanent incisors in children in southern Turkey: a retrospective study. Dent Traumatol.

[bib-002] Andreasen JO, Andreasen FM, Andreasen JO, Andreasen (1993). Avulsion. Textbook and Colour Atlas of Traumatic Injuries to the teeth.

[bib-003] Sanu OO, Utomi IL (2005). Parental awareness of emergency management of avulsion of permanent teeth of children in Lagos, Nigeria. The Nig Postgrad Med Journ.

[bib-004] Marin PD (2000). The avulsed tooth - the best implant. Ann R Australas Coll Dent Surgery.

[bib-005] Chan AWK, Wong TKS, Cheung GSP (2001). Lay knowledge of physical education teachers about the emergency management of dental trauma in Hong Kong. Dent Traumatol.

[bib-006] Piccin A, Fleming P, Eakins E, McGovern E, Smith OP, McMahon C (2008). Sickle cell disease and dental treatment. J Ir Dent Assoc.

[bib-007] Ramakrishna Y (2007). Dental considerations in the management of children suffering from Sickle cell disease: a case report. Indian Soc Pedod Prev Dent.

[bib-008] Duggal MS, Bedi R, Kinsey SE, Williams SA (1996). The dental management of children with sickle cell disease and beta-thalassaemia: a review. Int J Paediatr Dent.

[bib-009] Sonis ST (2004). Orthodontic management of selected medically compromised patients: cardiac disease, bleeding disorders, and asthma. Semin Orthod.

[bib-010] Utomi IL, Sanu OO, Obisesan BA, Aluko IA, Isiekwe MC (2003). Replantation of Avulsed Permanent Teeth: A case report. Nig J Health 7 Biomed Sci.

[bib-011] Adekoya-Sofoluwe C, Sote EO, Odusanya S, Fagade O (2000). Traumatic dental injuries of anterior teeth of children in Ile-Ife. Paediatric Dent Journ.

[bib-012] Skaare AB, Jacobsen I (2003). Dental injuries in Norwegians aged 7-18 years. Dental Traumatol.

[bib-013] Zerman N, Cavalleti G (1993). Traumatic injuries to permanent incisors. Endod Dent Traumatol.

[bib-014] Onetto JE, Flores MT, Garbarino ML (1994). Dental trauma in children and adolescents in Valparaiso, Chile. Endod Dent Traumatol.

[bib-015] Trope M, Chivian N, Sigurdsson A, Vann WF, Cohen S, Burns RC (2002). Traumatic injuries. Pathways of the Pulp.

[bib-016] Alves PVM, Alves DKM, de Souza MMG, Torres SR (2005). Orthodontic Treatment of Patients with Sickle-Cell Anemia. The Angle Orthodontist.

[bib-017] Arowojolu MO, Savage KO, Aken’ova YA (1996). Periodontal disease in homozygous HBSS adolescent Nigerians. Afr J Med Med Sci.

[bib-018] Crawford JM (1988). Periodontal disease in sickle cell disease subjects. J Periodontol.

